# A missing piece in Bangladesh's nutrition agenda: indigenous children and adolescents in the Chittagong Hill Tracts

**DOI:** 10.1016/j.lansea.2025.100685

**Published:** 2025-10-25

**Authors:** Satyajit Kundu, Rajat Das Gupta

**Affiliations:** a ARCED Foundation, Dhaka, Bangladesh; b Public Health, School of Medicine and Dentistry, Griffith University, Gold Coast, Queensland, Australia; c BRAC James P Grant School of Public Health, BRAC University, Dhaka, Bangladesh; d Division of Epidemiology, Department of Medicine, Vanderbilt University Medical Center, Nashville, TN, USA

Bangladesh has made strong progress in improving child survival and reducing malnutrition over the past two decades.^1^ However, not all communities have shared in this success. Indigenous children and adolescents living in the Chittagong Hill Tracts (CHT), a remote, hard-to-reach, hilly region in the southeast of the country, continue to face persistent health and nutrition inequalities^2^ that places them at serious risk.

The CHT is home to over a million Indigenous people from eleven ethnic communities, most living in rural areas with limited access to healthcare, education, clean water, and food.^3^, ^4^, ^5^ Our systematic literature review identified only a handful of studies, all showing that Indigenous children in Bangladesh experience worse nutrition outcomes than children in the general population. For example, Haque and Islam^6^ reported that in 2018, more than 30% of Indigenous preschool children in Bandarban were underweight, around 45% were stunted, and 15% were wasted, compared with national averages in 2017–18 of 22% underweight, 31% stunted, and 8% wasted among children under 5 years.^7^ A survey reported that only 12–13% of Marma and Mro children (2 ethnic communities) in Bandarban achieved minimum dietary diversity, defined by WHO as consumption of at least four of seven food groups in the past 24-h, compared with more than 50% among Bengali children in the Bandarban district of CHT.^8^

A national survey by the United Nations Children's Fund included a few indigenous children and also revealed that Indigenous children are more likely to experience severe wasting, stunting, and inadequate meal frequency compared to their non-Indigenous peers.^9^ Critical factors, such as breastfeeding practices and the nutritional status of women during pregnancy, directly affect the nutritional outcomes of young children. It is reported that the rate of early initiation of breastfeeding among children in the Bandarban district of CHT in 2017 was 37%,^10^ which is much lower than the national average of 60% in 2017–18,^7^ reflecting suboptimal practices. Yet comprehensive data on the nutritional status of Indigenous women during pregnancy in CHT remain scarce. Moreover, the existing studies are fragmented, small-scale and limited in scope, and descriptive, leaving a critical gap in comprehensive assessments.

These early disadvantages don't end in childhood. As children grow into adolescence, the nutritional challenges continue. Nationally, most adolescents in Bangladesh consume diets low in fruits and vegetables and high in unhealthy foods like sugary drinks and fried snacks.^11^ Among Indigenous adolescents, although less studied, the situation is likely worse because their dietary patterns could be exacerbated by additional barriers: geographic isolation, marginalisation from mainstream education and health systems, poor food access, and erosion of traditional food practices linked to land dispossession and conflict.^3^^,^^4^ In addition, limited knowledge about healthy eating, low school attendance, and minimal exposure to nutrition education programs collectively may further reduce diet quality and increase the risk of long-term health problems in adulthood.^2^^,^^5^

Despite these urgent needs, Indigenous children and adolescents are rarely included in national nutrition policies. Existing efforts by non-government organisations (NGOs) and donor programs often focus on maternal nutrition while overlooking children and adolescents, and fail to comprehensively assess their dietary practices, nutritional status, or the unique risk factors of their diet and nutritional status. Indigenous adolescents in particular have been almost entirely neglected, despite being a critical transitional period and stage for breaking intergenerational cycles of malnutrition.^12^ The lack of disaggregated data on their diet, nutrition, and health behaviours further makes it harder to plan effective, evidence-based actions, leaving policymakers blind to these needs and rendering them invisible in national strategies.

Without timely action, the current neglect of Indigenous children and adolescents will only reinforce cycles of malnutrition, poverty, and poor health across generations. Although Bangladesh's Eighth Five-Year Plan and the National Strategy for Adolescent Health mention Indigenous populations, operational guidance, investment, and programmatic inclusion remain minimal. Bangladesh's commitment to the Sustainable Development Goals cannot be achieved without addressing the specific needs of Indigenous children and adolescents. There is an urgent need to generate reliable and culturally appropriate comprehensive data on their diet quality and nutritional assessment. Future research should therefore prioritise large-scale disaggregated surveys, qualitative studies on cultural food practices and barriers, and community-driven programs tailored to both children and adolescents in CHT, to provide the evidence base for effective policies and interventions.

National health strategies must explicitly prioritise Indigenous communities, and nutrition programs should be designed with active participation from Indigenous youth and community leaders to ensure cultural appropriateness, drawing on local food systems, beliefs, and languages. Nutrition education tailored to children and adolescents should be delivered through schools and communities in culturally appropriate ways. Furthermore, greater coordination among government agencies, NGOs, and donors is essential to ensure that nutrition policies and programs are inclusive and sustainable. These steps are vital to ensure that Indigenous children and adolescents are no longer left behind and to achieve equitable progress in health and nutrition in Bangladesh.

## Contributors

**Satyajit Kundu:** Conceptualisation, literature search, data interpretation, writing of the manuscript, critical revisions, and validation.

**Rajat Das Gupta:** Writing of the manuscript, critical revisions, and validation.

## Declaration of interests

None declared.
